# Human Cell-Based *in vitro* Phenotypic Profiling for Drug Safety-Related Attrition

**DOI:** 10.3389/fdata.2019.00047

**Published:** 2019-12-11

**Authors:** Ellen L. Berg

**Affiliations:** Eurofins Discovery, Translational Biology, Burlingame, CA, United States

**Keywords:** drug discovery, phenotypic assays, toxicity mechanism, reference database, data mining, toxicity testing, new approach methodologies

## Abstract

Ensuring the safety of new drugs is critically important to regulators, pharmaceutical researchers and patients alike. Even so, unexpected toxicities still account for 20–30% of clinical trial failures, in part due to the persistence of animal testing as the primary approach for de-risking new drugs. Clearly, improved methods for safety attrition that incorporate human-relevant biology are needed. This recognition has spurred interest in non-animal alternatives or new approach methodologies (NAMs) including *in vitro* models that utilize advances in the culture of human cell types to provide greater clinical relevance for assessing risk. These phenotypic assay systems use human primary and induced pluripotent stem cell-derived cells in various formats, including co-cultures and advanced cellular systems such as organoids, bioprinted tissues, and organs-on-a-chip. Despite the promise of these human-based phenotypic approaches, adoption of these platforms into drug discovery programs for reducing safety-related attrition has been slow. Here we discuss the value of large-scale human cell-based phenotypic profiling for incorporating human-specific biology into the de-risking process. We describe learnings from our experiences with human primary cell-based assays and analysis of clinically relevant reference datasets in developing *in vitro*-based toxicity signatures. We also describe how Adverse Outcome Pathway (AOP) frameworks can be used to integrate results from diverse platforms congruent with weight-of-evidence approaches from risk assessment to improve safety-related decisions in early discovery.

## Introduction

The use of “omics” profiling technologies (genomics, proteomics, metabolomics, etc.) and *in vitro* methods in discovery toxicology has exploded in recent years and begun to advance our understanding of toxicity mechanisms (Beilmann et al., 2019; Thomas et al., 2019). The field is still young, however, as the molecular mechanisms underlying most human toxicities remain unknown. Despite this, identified mechanisms reveal critical differences between humans and animals commonly employed in safety tests (Jang et al., 2019). In addition to the well-appreciated differences in gene and protein sequences, important influences of lifespan, environment, and physical architecture on toxicity mechanisms are less often recognized and addressed.

Lifespan: Whereas humans live for many decades (~70 years), rodents are short lived (~2 years) with comparatively limited regulatory mechanisms supporting genome maintenance and integrity. Indeed, DNA mutation rates are higher in mice than humans (Milholland et al., 2017) and expression of DNA repair genes is lower (MacRae et al., 2015). Humans also appear to be more reliant on immune system surveillance to identify and eliminate cells containing genomic mutations (Corthay, 2014). These lifespan-related differences in DNA damage response mechanisms are important to consider when assessing the relevance of resource-intense and costly two-year animal carcinogenicity studies, often performed for new product regulatory submissions (Corvi et al., 2017).

Environment: Environmental factors contribute to the many differences in immune system function described between humans and research animals (Mestas and Hughes, 2004; Seok et al., 2013). Humans live in the open world exposed to a diverse array of chemical, microbial, and infectious stressors over an extended timeframe. In contrast, research animals are housed in small groups in highly standardized, clean facilities with filtered water and specialized food. Indeed, immune mechanisms appear to drive a significant fraction of human-specific toxicity susceptibility and are associated with increased risk of adverse effects to drug exposure, including idiosyncratic liver toxicity (Godoy et al., 2013). Cytokine release syndrome, the tragic outcome in 2006 of the Phase I clinical trial of the CD28 super-agonist antibody TGN 1412, was an unpredicted immune-system toxicity in humans not observed in preclinical testing (Lee et al., 2014).

Physical Architecture: Toxicities are also influenced by species-specific tissue and organ architecture—for example the vascular system. Vascular beds in rodents are densely arranged, with characteristically short distances between the average tissue resident cell and the closest blood vessel (Karbowski, 2011; Brissova et al., 2015). In contrast, the vasculature in human tissues is less dense and contains larger vessels that are subject to significantly higher vascular wall shear stresses. Humans are more vulnerable than rodents to blocks in blood vessel flow (heart attacks, pulmonary thrombosis, stroke) and as a consequence, manifest additional regulatory mechanisms to control hemostasis. Dogs, with a higher degree of similarity to humans with regard to cardiovascular system architecture, are often employed in cardiovascular toxicity studies. These costly studies, however, have had limited impact on reducing safety attrition. Cardiovascular system-related toxicities continue to be one of the leading causes of clinical failures (Cook et al., 2014), emphasizing a critical need for NAMs that reflect human-specific vulnerabilities.

Translational approaches that capture the biology and mechanisms relevant to cell proliferation, immune, and inflammatory responses and vascular biology are well placed to reveal human-specific toxicity impacts. For these data to be utilized effectively, organizational strategies that integrate data from multiple platforms and capture mechanistic relationships are needed. This in turn can expedite adoption of human-based *in vitro* assays for safety de-risking in discovery.

## Adverse Outcome Pathway Frameworks for Integrating Assay Platform Results

**Figure 1:**
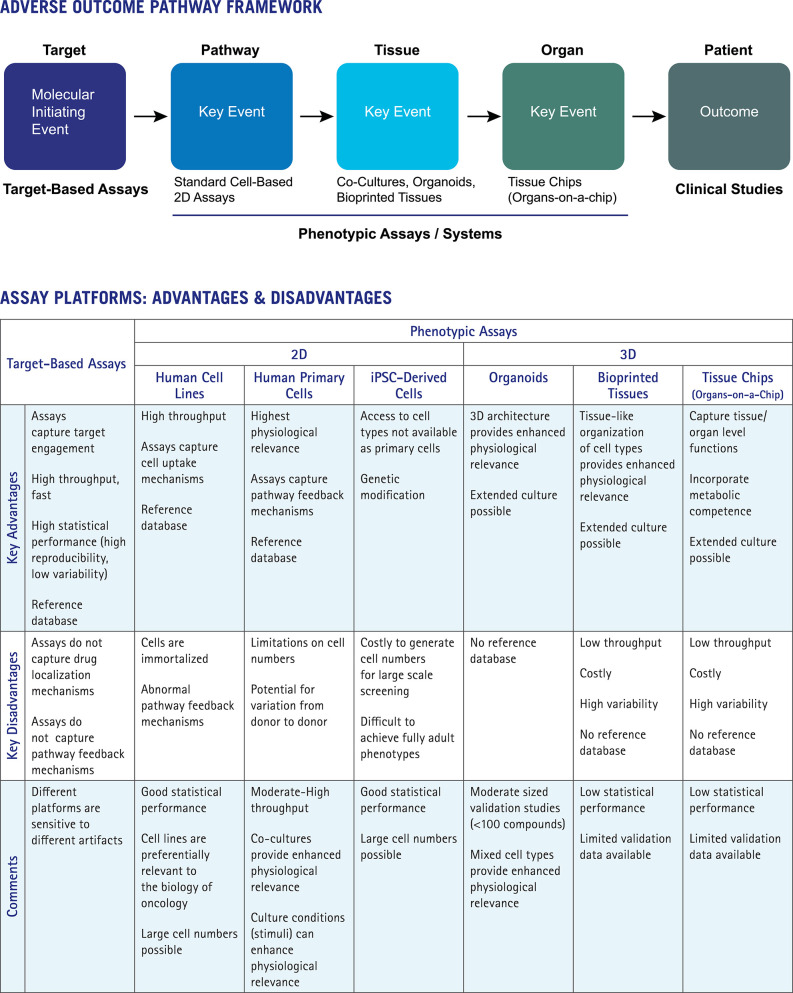
Mapping assay platforms to the Adverse Outcome Pathway framework.

The Adverse Outcome Pathway (AOP) approach provides a particularly useful framework as it aligns platforms and data types to key mechanistic steps. The use of AOPs to organize knowledge in support of toxicity risk assessments has been advanced by the OECD and the US EPA (Edwards et al., 2016; https://www.oecd.org/chemicalsafety/testing/adverse-outcome-pathways-molecular-screening-and-toxicogenomics.htm). AOPs are applied in a weight-of-evidence approach for risk assessment and used to connect molecular initiating events (e.g., target interactions) to adverse outcomes through a series of key events at distinct levels of the biological hierarchy: pathway, tissue, organ, organism, population. Figure 1 shows how the AOP framework can be used to integrate data from target-based platforms, phenotypic assays, more advanced complex systems, and clinical studies. Assay platforms map to different elements of the AOP framework, with important advantages and disadvantages of each approach indicated.

Target-based profiling platforms map to the first step in the AOP, the molecular initiating event (MIE). Broad profiling across diverse targets is a long-established approach for de-risking lead compounds (Bowes et al., 2012). Key advantages of target-based profiling include throughput for testing large numbers of compounds and statistical robustness of the resulting data, enabling quantitative differentiation of close analogs. Assays tend to be simple and fast with comparatively limited numbers of experimental variables. The disadvantages of target-based profiling include the limited numbers of targets available, restrictions on modes of action covered (e.g., some platforms do not differentiate between activators and inhibitors, or capture allosteric mechanisms), and potential for assay interference (i.e., frequent hitters, fluorescent compounds). Most importantly, target-based platforms simply do not capture many of the regulatory mechanisms controlling the outcome of drug-target interactions *in vivo*, such as pathway feedback mechanisms or processes that dictate drug concentration and localization in target tissues (e.g., ADME properties of absorption, deposition, metabolism and excretion).

Standard cell-based (e.g., 2D) assays map to the next step of the AOP framework, the pathway level. These assays capture some mechanisms relevant to *in vivo* activity including cell uptake and the ability to assess target function in the presence of intracellular pathway feedback mechanisms, depending on culture conditions. Feedback mechanisms relevant to human responses are preferentially retained by human primary or stem-cell-derived cells. These cell types more closely reflect cell behaviors in human tissues than do immortalized cell lines. Phenotypic 2D assays are scalable, with moderate to high throughput, and can achieve reasonably good statistical performance. These features are key for development of reference databases and analytical tools to run the comparative analyses that provide important mechanistic interpretation of phenotypic assay results. Disadvantages of 2D format assays include limited culture periods and lack of physiologically relevant 3D architecture and competence for drug metabolism. Past efforts have used individual organ-specific cell types (e.g., cardiomyocytes or hepatocytes) to predict organ-specific toxicity, with cell function or cytotoxicity as proxy endpoints (Godoy et al., 2013; Dragovic et al., 2016; Magdy et al., 2018). The performance of these assays in predicting organ-specific toxicity (hepatocytes for liver toxicity; cardiomyocytes for cardiovascular system toxicity) has been unsatisfactory as organ toxicity can be caused by a number of mechanisms involving additional cell types, such as immune cells or the vasculature. Limited performance of these assays (Dragovic et al., 2016) has led researchers toward more advanced cellular systems that incorporate diverse cell types and capture complex aspects of tissue and organ-level biology.

Organoids, bioprinted tissues, and organs-on-a-chip represent advanced cellular systems that incorporate additional physiologically relevant features such as 3D architecture, metabolic competence, and tissue level functions (Horvath et al., 2016). The key advantage of these platforms is the ability to reflect higher level tissue functions, including drug metabolism and its impacts. Therefore, these approaches map to the tissue and organ levels of the AOP (Figure 1). While these systems hold promise in safety de-risking, confidence in their use and adoption into the drug discovery process has lagged, tempered by lack of throughput and limited validation data. Low assay throughputs (whether technical or economical) preclude development of reference standards databases—large scale data sets are needed to truly assess platform robustness, assay reproducibility, and data predictivity. Scale and throughput have advanced somewhat for organoids and 3D cultures with interesting emerging results, albeit with only moderately sized data sets (Sirenko et al., 2019). As the complexity of advanced cellular systems grows with larger numbers of experimental variables and longer culture times, data variability inevitably increases and may become the limiting factor in their utility and adoption. Without capabilities for large scale comparative analyses, these systems and platforms provide limited mechanism of action, so may best support further exploration of well-characterized drug leads already tested for target selectivity in large panels and evaluated for effects on diverse cellular pharmacology.

## Phenotypic Profiling for Human-Relevant Toxicity Mechanisms

In isolation, no single phenotypic assay or platform provides sufficient predictive power for safety-related decisions. Integrating information from diverse sources in a weight-of-evidence approach for assessing risk is needed. As touched on above, each assay technology has advantages and disadvantages, including applicability domains, statistical and reproducibility limitations, validation to clinical results, reference database availability for data mining and analytics, and sensitivity to artifacts. However, phenotypic platforms that can connect target mechanisms to tissue context may be preferentially useful.

The BioMAP® platform of human primary cell-based systems (Eurofins Discovery) was successfully used by the EPA for the ToxCast™ program [(Houck et al., 2009; Kleinstreuer et al., 2014a) and data available through the website: https://comptox.epa.gov/dashboard], and by various pharmaceutical researchers for characterization of new drug leads (reviewed in Berg, 2017). Systems of the BioMAP platform represent a broad range of tissue biology (vascular, respiratory, immune, tumor microenvironment and skin) in individual and co-culture formats; protein biomarkers with established clinical annotations serve as measured translational biology endpoints. The platform addresses biology relevant to human-specific toxicity mechanisms and includes proliferation endpoints to enable detection of cell type selective mechanisms, particularly important for assessing the safety of oncology drugs where discrimination between tumor and normal host cells is critical for minimizing adverse effects. Systems covering biology of the vasculature incorporate endothelial cells and vascular smooth muscle cells, and studies using these assays have led to the discovery of a novel toxicity mechanism underlying drug-induced thrombosis related side effects in humans (Berg et al., 2015). Limitations of the BioMAP platform include a lack of 3D architecture, relatively short culture times (1–6 days), and limited capabilities for drug metabolism. Notwithstanding, the format and scale of this platform offers significant advantages over more complex advanced cellular systems. In particular, the level of reproducibility and throughput has provided for generation of a large-scale reference database and development of analytics for assigning mechanism of action (Berg, 2017).

Comprehensive databases, given reasonable levels of assay reproducibility and variability, allow comparative analyses to reference standards and mechanism of action distinctions. The BioMAP Reference Database contains biomarker profiles generated from >4,500 test agents including drugs, experimental chemicals, extracts, and environmental chemicals, enabling mechanism of action classification development of predictive toxicity signatures. Large-scale databases exist for other phenotypic platforms including: the database of drug sensitivities across panels of cancer cell lines (Seashore-Ludlow et al., 2015; and the Cancer Therapeutics Response Portal CTRP, https://portals.broadinstitute.org/ctrp/); the database of gene expression profiles containing a subset of genes using the L1000 platform (Subramanian et al., 2017); the NIH LINCS program portal for Library of Integrated Network-Based Cellular Signatures, http://www.lincsproject.org/LINCS/); and a database of images from high content imaging assays using multiplexed fluorescent dyes (Cell Painting, Bray et al., 2017). Given sufficient data and statistical power, phenotypic assays can distinguish diverse mechanisms of action, with prediction accuracy increasing with increasing size of the database coverage.

## Development of Toxicity Signatures

Recently, BioMAP data from drugs and experimental therapies tested in humans has been applied to identify phenotypic signatures (toxicity signatures) associated with particular clinical adverse effects (e.g., Berg et al., 2015; Shah et al., 2017). BioMAP profiles for a small set of key reference drugs associated with a particular clinical adverse effect (AE or adverse outcome) were analyzed to identify shared biomarker activities among drugs associated with the same AE. Common activities were investigated for their potential to contribute to a toxicity signature by evaluating the full BioMAP Reference Database for all agents exhibiting the signature and determining the level of association of these agents with the clinical AE. Successful signatures were further developed if reference agents were found to be preferentially associated (>90%) with the clinical AE.

Deficiencies in reporting adverse effects do not permit reliable statistical performance metrics for predictive toxicity signatures. Without a statistically rigorous foundation, in order for these signatures to be of practical use, pathway mechanisms driving these signatures are needed. To accomplish this, active reference agents from the database were investigated for their reported mechanisms of action. Target mechanisms specifically enriched in any given reference group were then assigned to the toxicity signature. Following this approach over the past decade, nine human toxicity signatures were developed: acute toxicity, organ toxicity, immunosuppression, liver toxicity, skin rash, irritation, sensitization, thrombosis-related side effects, and vascular inflammation. Table 1 shows the list of signatures, key biomarkers, representative drugs, associated mechanisms and reference publications.

**Table 1:** List of toxicity signatures with key reference compounds, biomarkers and associated pathway and target mechanisms.

**Toxicity signature**	**Definition**	**Key reference compounds**	**Key biomarker**	**Pathway/Target mechanisms**	**References**
Acute toxicity	Increased risk of death	A23187, monensin, cycloheximide, actinomycin D, digoxin, bortezomib, valinomycin; Classes: cardiac glycosides, alkylating agents, ionophores	Decreased SRB in multiple systems (five biomarkers in signature)	Protein synthesis inhibition, RNA synthesis inhibition, Na+/K+ ATPase dysfunction	Houck et al., 2009; Berg, 2017
Immuno-suppression	Increased risk of infection	Sirolimus, infliximab, cyclosporine, tacrolimus, mycophenolate, azathioprine	Decreased T cell proliferation in BioMAP SAg system (five biomarkers in signature)	mTOR, calcineurin, JAK, Hsp90, NFAT, DNA proliferation	Berg, 2017
Skin irritation	Irritation of the skin with reddening and itch	Retinoic acid, retinol, vitamin D, ritonavir, imatinib, 2-chloroethyl ethyl sulfide, calcitriol; Classes: retinoids, vesicants, blister agents	Increased PGE2 in BioMAP LPS system (three biomarkers in signature)	RAR/RXR, AhR, VDR receptor classes	Shah et al., 2017
Liver toxicity	Increased incidence of hepatotoxicity, liver steatosis	Amiodarone, tamoxifen, astemizole, ketoconazole, haloperidol, aplaviroc	Decreased SRB in BioMAP 3C system (four biomarkers in signature)	V-ATPase, PIKfyve, smoothened	Shah et al., 2014
Organ toxicity	DNA replication-related organ toxicity	5-fluorouracil, vincristine, monensin, cisplatin; Classes: chemotherapeutic agents, anti-metabolites, anti-mitotics	Decreased proliferation in BioMAP 3C system (three biomarkers in signature)	DNA replication, microtubules	Berg, 2017
Skin rash (MEK-related)	Increased likelihood of acniform skin rash	Trametinib, AZD6244, p38 MAPK inhibitors, Betaseron®, anakinra	Increased VCAM-1 in BioMAP HDF3CGF system (two biomarkers in signature)	MEK, p38 MAPK, IL-1R, IL-4R, Tweak receptor, IFNα/β	Kleinstreuer et al., 2014a
Skin sensitization	Increased potential for allergic skin reactions	NSAIDS, phthalates	Decreased Collagen III in BioMAP HDF3CGF system (two biomarkers in signature)	RAR/RXR, PKC, mitochondria, JNK, prostaglandin receptor	Kleinstreuer et al., 2014b
Thrombosis	Increased incidence of stroke, deep vein thrombosis, pulmonary embolism	Sirolimus, crizotinib, raloxifene, tamoxifen, clozapine; Classes: polycyclic aryl hydrocarbons, selective estrogen receptor modulators, anti-psychotics	Increased TF/CD142 in BioMAP 3C system (three biomarkers in signature)	mTOR, AhR, V-ATPase, lysosomal function, CYP17A, PKC, NOD2, HIF-1α and receptors for estrogen, histamine H1, thyroid hormone and oncostatin M	Berg et al., 2015
Vascular toxicity	Increased incidence of hypertension, cardiovascular events due to atherosclerosis	Prednisolone, budesonide, panobinostat, trametinib, nicotine	Increased SAA in BioMAP CASM3C system (two biomarkers in signature)	MEK, glucocorticoid and mineral corticoid receptors, HDAC, IL-6R, NAD+/NADH ratio	Berg, 2017

## Application of Toxicity Signatures in Investigative Toxicology and Early Discovery

Predicting the likelihood of drug-induced toxicities in humans is challenging due to known limitations of drug side effect data (Maciejewski et al., 2017). The study from Shah et al. (2017) describes the application of toxicity signatures for investigative toxicology. Researchers at Pfizer previously discovered that a preclinical lead for an mGluR5 program caused skin toxicity in non-human primates, but not in rodents (Zhang et al., 2014). This compound was evaluated in the BioMAP Diversity PLUS® Panel, and analysis of the profile data indicated presence of the BioMAP Toxicity Signature for skin irritation (see Table 1, Definition). Data mining previously associated this signature with activity against the nuclear hormone receptor (NHR) targets, RAR/RXR (agonism), AhR (antagonism), or VDR (agonism). Using an orthogonal assay platform (Attagene Factorial® technology, Morrisville NC), the lead compound was confirmed to have activity in functional assays for AhR and VDR, two of the mechanisms previously associated with the skin irritation signature. Additional identified T-cell response biomarker activities supported a proposed hypothesis to explain the observed T cell-mediated type IV hypersensitivity reaction.

This case study illustrates the importance of combining results from multiple platforms to support program advancement. Indeed, the NHR assays highlighted above are not included in standard safety pharmacology panels, as these have not shown sufficient predictivity for safety-related outcomes. Only when combined with the phenotypic profiling results, which provides both tissue context and potential clinical biomarkers, can these assays be useful to a program. Together the results provide sufficient mechanistic rationale to explain why the skin toxicity was not seen in lower animal species and to develop methods for assessing this mechanism in clinical studies.

## Discussion

Adopting the AOP framework for integrating information from diverse platforms can help drive acceptance of phenotypic assay NAMs by facilitating their incorporation into weight-of-evidence decision strategies. AOPs emphasize the value of information as representative of a key event modality, rather than as an isolated result from a single assay or platform. AOPs can also accommodate other important information for safety de-risking such as *in silico* models and other *in vitro* data for predicting pharmacokinetics and ADME properties. These are critical for predicting toxicity effects *in vivo* and for quantifying safety risks. Indeed, quantitative AOP models represent an active area of research (Perkins et al., 2019; Saili et al., 2019). Translation of results from animal studies is also facilitated as each of the key mechanistic steps can be evaluated and compared across species. Understanding human/animal differences at the mechanism level facilitates the selection of appropriate animal models and incorporation of study results into program decisions.

Future research in this area will see advanced complex systems continue to improve and scale. Indeed, new systems for modeling the CNS, kidney, and cancer have been recently described (Petrosyan et al., 2019; Plummer et al., 2019; Trujillo et al., 2019). Key challenges remain, however, particularly in data management and analysis. Assay systems can be complicated requiring significant metadata to describe them effectively. In addition, there has been relatively little standardization around phenotypic data analysis methods. This has made it difficult to compare data across platforms and to make results easily interpreted. Solutions in these areas to standardize analysis methods, incorporate metadata ontologies, and enable data integration, perhaps in conjunction with new methods for data visualization, will be important to facilitate adoption of NAMs. Human-based *in vitro* methods, and in particular innovative NAMs, have the potential to be especially helpful in reducing safety-related attrition in drug development. They can provide the necessary context at the appropriate pathway, cellular, tissue, or organ level for understanding toxicity mechanisms. Platforms that have sufficient scale and reproducibility to generate large reference databases are particularly useful as they provide a relational connection between targets and clinical outcomes. Anchoring phenotypic NAMs to a mechanistic framework such as those provided by the AOP construct will help with adoption of these approaches, as toxicology moves away from a descriptive science to an evidence-based, mechanistic discipline (Beilmann et al., 2019).

## Author Contributions

EB conceived of and wrote this perspective.

### Conflict of Interest

EB is an employee of Eurofins Discovery which provides phenotypic profiling services, some of which are described in the present manuscript, and therefore has a potential conflict of interest to declare.
